# Evaluation of the Initial General Ward Early Warning Score and ICU Admission, Hospital Length of Stay and Mortality

**DOI:** 10.5811/westjem.2021.6.50657

**Published:** 2021-09-02

**Authors:** Anneke Gielen, Kristine Koekkoek, Marijke van der Steen, Martijn-Looijen, Arthur R.H. van Zanten

**Affiliations:** * Gelderse Vallei Hospital, Department of Intensive Care Medicine, Ede, The Netherlands; † Gelderse Vallei Hospital, Department of Information Technology and Datawarehouse, Ede, Netherlands; ‡ Wageningen University & Research, Division of Human Nutrition and Health, Wageningen, The Netherlands

## Abstract

**Introduction:**

Despite widespread implementation of the Early Warning Score (EWS) in hospitals, its effect on patient outcomes remains mostly unknown. We aimed to evaluate associations between the initial EWS and in-hospital mortality, intensive care unit (ICU) admission, and hospital length of stay (LOS).

**Methods:**

We performed a retrospective cohort study of adult patients admitted to a general hospital ward between July 1, 2014–December 31, 2017. Data were obtained from electronic health records (EHR). The primary outcome was in-hospital mortality. Secondary outcomes were ICU admission and hospital LOS. We categorized patients into three risk groups (low, medium or high risk of clinical deterioration) based on EWS. Descriptive analyses were used.

**Results:**

After applying inclusion and exclusion criteria, we included 53,180 patients for analysis. We found that the initial (low- vs high-risk) EWS was associated with an increased in-hospital mortality (1.5% vs 25.3%, P <0.001), an increased ICU admission rate (3.1% vs 17.6%, P <0.001), and an extended hospital LOS (4.0 days vs 8.0 days, P <0.001).

**Conclusion:**

Our findings suggest that an initial high-risk EWS in patients admitted to a general hospital ward was associated with an increased risk of in-hospital mortality, ICU admission, and prolonged hospital LOS. Close monitoring and precise documentation of the EWS in the EHR may facilitate predicting poor outcomes in individual hospitalized patients and help to identify patients for whom timely and adequate management may improve outcomes.

## INTRODUCTION

Early identification and management of critically ill adult patients admitted to general hospital wards may prevent in-hospital mortality and unplanned intensive care unit (ICU) admission and decrease hospital length of stay (LOS).^1^–^3^ Several hours before ICU admission or cardiopulmonary arrest, changes in vital signs can be detected by medical and nursing staff.^3^–^6^ However, poor monitoring, misinterpretation of vital signs, and inadequate management by the clinical staff may contribute to “preventable” adverse events.^2^,^3^,^7^ To systematically monitor vital signs and recognize deteriorating patients in a timely fashion, Early Warning Score (EWS) systems have been developed. These systems are established to detect alarm signals (eg, hypoxia, hypotension, tachycardia, tachypnea, and changes in mental function) and thereby predict and prevent adverse events. The EWS is a simple-to-use bedside tool that helps to identify the critically ill patient at risk of acute clinical deterioration.^1^,^2^,^8^ These track-and-trigger systems use an algorithm that allocates points based on abnormal physiological variables.

When the cumulative EWS reaches certain thresholds, it triggers a specific response, eg, more frequent monitoring, notification of the ward doctor, and/or a consult by a rapid response team (RRT).^1^,^2^ The purpose of an RRT is to provide early and adequate management of clinically deteriorating patients in general hospital wards.^9^ Despite the widespread implementation of RRT and EWS systems, the available evidence of the effect of these interventions is limited and of poor quality.^2^,^9^–^11^ The Committee of Practice Guidelines Development of the Dutch Society of Intensive Care Medicine (Nederlandse Vereniging voor Intensive Care, NVIC) concludes that early intervention by an RRT may prevent unplanned ICU admissions. The conmittee recommends distributing a table with early warning criteria in the hospital for early identification of the deteriorating patient, and early consultation by the RRT.^12^ Gelderse Vallei Hospital introduced a RRT in 2008. The RRT is comprised of medical and nursing staff from the ICU.^13^

Our hospital has implemented an EWS to timely detect the clinically deteriorating patient and hence improve patient prognosis. However, evidence for the effect of these interventions on patient outcomes is limited, and its exact effect remains mostly unknown. Therefore, this study aims to evaluate the effect of an EWS on patient outcomes by addressing the associations between the initial EWS and in-hospital mortality, ICU admission, and hospital LOS.

## METHODS

This study was a retrospective, observational, single-center cohort study of medical and surgical patients admitted to a general hospital ward between July 1, 2014–December 31, 2017. We included all adult patients (≥ 18 years old) admitted to a general hospital ward with one or more recorded EWS. Exclusion criteria were as follows: EWS with more than three missing variables; patients discharged within 72 hours after being admitted to the emergency department or day treatment; and patients with elective ICU admission. Elective ICU admissions were considered unrelated to the EWS recorded on a general ward because of their routine nature and the decision to admit to the ICU for other reasons such as surgical procedures (ie, comparable to the post-anesthesia care unit). Therefore, elective ICU admissions were considered outside the scope of this study. The institutional review board of the Gelderse Vallei Hospital approved the study and waived informed consent for the retrospective design and anonymization of patient identifiers before analysis.

### Early Warning Score

**Figure 1:**
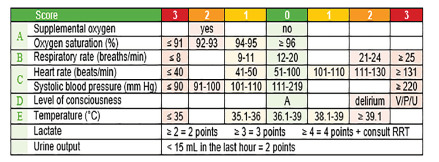
The seven standard variables plus two additional variables and point allocation for each variable. *A*, alert; *V*, response to voice; *P*, response to pain; *U*, unresponsive; *RRT*, rapid response team; *mL*, milliliter.

The EWS is comprised of seven standard variables and two additional variables (Figure 1). The seven standard variables are supplemental oxygen, oxygen saturation, respiratory rate, heart rate, systolic blood pressure, level of consciousness, and temperature. For each of these variables, 0–3 points are allocated based on their value. Extra points are allocated for two additional variables, lactate levels and urine output: high lactate (lactate ≥ 2 millimoles per liter (mmol/L), 2 extra points; lactate ≥ 3 mmol/L, 3 extra points; lactate ≥ 4 mmol/L, 4 extra points); and reduced urine output (urine output < 15 milliliters in the last hour, 2 extra points). The sum of these points is automatically generated by the electronic health record (EHR), resulting in the cumulative EWS. When the EWS reaches certain thresholds, it triggers subsequent actions executed by nursing and medical staff (eg, more frequent monitoring or a consult by the RRT).

**Population Health Research Capsule**

What do we already know about this issue?

*Despite widespread implementation of Early Warning Scores (EWS) and hospital rapid response teams, evidence of the effect on patient outcomes is limited*.

What was the research question?

*Is the initial, general ward EWS associated with ICU admission, hospital length of stay, and in-hospital mortality?*

What was the major finding of the study?

*An initial high EWS was associated with ICU admission, prolonged hospital stay, and high in-hospital mortality*.

How does this improve population health?

*Early EWS monitoring in general wards may facilitate predicting poor outcomes and identifying patients for whom timely management may improve outcomes*.

In the EWS system implemented by Gelderse Vallei Hospital, these thresholds are set at low risk (EWS 0–5), medium risk (EWS 6–8), and high risk (EWS ≥ 9) of clinical deterioration. Per common practice, nurses check the vital signs of patients at the general hospital wards routinely once every eight hours. In cases where the EWS remains 0–2, this frequency could be reduced to once every 12–24 hours after consulting the ward physician.^15^ A mildly elevated low risk (EWS 3–5) or feelings of concern (ie, a sense of alarm) perceived by the nurses requires the nursing staff to check the vital signs once every four hours and to consult the ward physician.

A medium-risk EWS (EWS 6–8) requires the nursing staff to check the vital signs at least once every one to two hours, to perform an arterial blood gas analysis (including lactate), and to consult the ward physician. In case of a medium-risk EWS the ward physician needs to assess the patient within 30 minutes of consultation. A high-risk EWS (EWS ≥ 9) requires blood gas analysis (including lactate) and immediate consultation of the ward physician. In case of a high-risk EWS the ward physician must assess the patient within 15 minutes of consultation and call the RRT.

### Outcomes

We categorized the initial EWS scores into low-risk (EWS 0–5), medium-risk (EWS 6–8), and high-risk (EWS ≥ 9) groups, and non-categorized (EWS 0–20). The primary outcome was in-hospital mortality. Secondary outcomes were unplanned ICU admission and hospital LOS. We subcategorized the outcome measure “unplanned ICU admission” into code status upon admission to a general ward. This subanalysis was performed because a negative ICU code status (not to be admitted to the ICU) could be a strong confounder in case of a high-risk EWS, causing a spurious association between the high-risk EWS and unplanned ICU admission. We performed a second subanalysis on all patients with a high-risk EWS who were not admitted to the ICU, despite a positive ICU code status (to be admitted to the ICU).

### Data Collection

We performed data extraction using SAS Enterprise Guide queries (SAS Institute, Inc., Cary, NC). All data were obtained from the EHR. Registered nurses monitored and documented the EWS in the EHR. The first 50 serial recorded EWS in the first two weeks of admission were included in this database for analysis. Baseline characteristics included gender, age, admission type (medical or surgical), code status, and RRT consultation. The code status upon admission was registered. We categorized code status into full code (cardiopulmonary resuscitation and intubation if required, ie, positive ICU code status); Do Not Resuscitate [DNR], ie, positive ICU code status); or Do Not Resuscitate/Do Not Intubate (DNR/DNI, ie, negative ICU code status).

The initial EWS was defined as the first EWS recorded for each patient upon admission to a general ward. We extracted the date of death from our electronic patient management system, which is connected to the municipal registration system. The patient was presumed alive if no date of death was registered. In-hospital mortality was defined as death during hospital admission. Elective ICU admission was defined as routine ICU admission, eg, after major surgery, while unplanned ICU admission was defined as an unanticipated transfer to the ICU during hospital admission.^14^ In the event of an ICU admission, RRT consultation was assumed according to standard practice in our hospital, and missing data of the RRT consultation were interpreted as incomplete registration. Days were defined as calendar days. We assessed the quality of the EWS database. Missing data were defined as empty cells or non-numerical data. We defined false entries as extreme values that were found to be highly implausible or outright impossible. Values with one or more decimal places for oxygen saturation, respiratory rate, heart rate, systolic blood pressure, and consciousness level were considered false entries.

### Data and Statistical Analysis

We report descriptive data as frequencies and percentages or ranges (minimum–maximum), means and standard deviation for data with a normal distribution or median, and first and third quartile [Q1–Q3] for data with a skewed distribution. The Kolmogorov-Smirnov test was used to test for normality. We assessed differences in baseline characteristics and outcomes with a chi-square test or a Fisher’s exact test, and a one-way analysis of variance (ANOVA) where appropriate. If ANOVA showed a significant difference, we applied a Tukey post-hoc test to detect differences between risk categories. A *P*-value of less than 0.05 was considered statistically significant. All statistical analyses were performed using IBM SPSS Statistics for Windows, version 24.0 (IBM Corporation, Armonk, NY).

## RESULTS

### Baseline Characteristics of the Study Population

**Figure 2:**
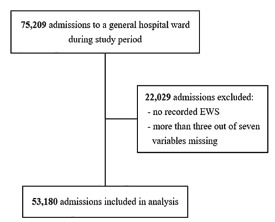
Flowchart of the study population.

During the study period, 75,209 adult patients were admitted to a general hospital ward. We excluded 22,029 patient admissions (29.3%) because no EWS was recorded or more than three of the seven standard variables were missing (Figure 2). In total, 53,180 admissions were included for further analysis.

The baseline characteristics of the study population are shown in Table 1. The final study population consisted of 53,180 patient admissions, including 33,628 individual patients with a total of 457,184 recorded EWS. Patients were categorized into three EWS risk groups: low, medium, or high risk of clinical deterioration. The median age was 68 years (range, 18–105), and 28,233 patients (53.1%) were female. Of all patient admissions 19,343 (36.4%) underwent a surgical procedure, and 33,837 (63.6%) were non-surgical admissions. The code status upon admission was full code in 39,369 (74.0%); DNR in 5331 (10.0%); and DNR/DNI in 8480 patient admissions (15.9%). In 1081 patient admissions (2.0%), the code status changed at least once during hospitalization. We documented RRT consultation in 1400 (2.6%) of all admissions. Significant differences between the three risk groups were observed in all variables.

### Primary Outcome

**Figure 3:**
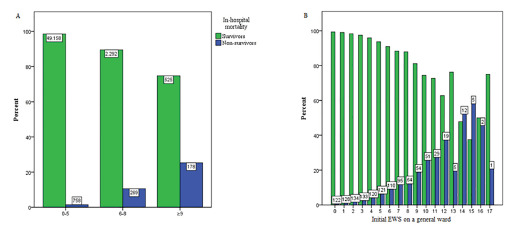
Association between the initial Early Warning Score (EWS) on a general hospital ward and in-hospital mortality rates. Non-survivors died during hospital admission. Survivors were discharged alive. Bars represent mortality or survival rates. Numbers represent the actual number of cases in the specific EWS category depicted. A) EWS categorized in low- (EWS 0–5), medium- (EWS 6–8) and high-risk (≥ 9) groups. B) EWS non-categorized.

The overall in-hospitality mortality was 2.3% (n = 1205), and 51,975 patients (97.7%) were discharged alive. A total of 758 (1.5%), 269 (10.5%), and 178 (25.3%) died during hospital admission in the low- (EWS 0–5), medium- (EWS 6–8) and high-risk (EWS ≥ 9) groups, respectively (Table 2). There was a statistically significant difference between the three risk groups (*P* <0.001). Figure 3 shows the association between the initial EWS on a general hospital ward (categorized into risk groups and non-categorized) and the in-hospital mortality compared to patients who were discharged alive. In general, for each point increase in the EWS the in-hospital mortality increased as well.

### Secondary Outcomes

Secondary outcomes for the three risk categories based on the initial EWS on a general ward are shown in Table 2. An elevated initial EWS was associated with an increased ICU admission rate (3.1% vs 17.6%, *P* <0.001) and an extended hospital LOS (4.0 days vs 8.0 days, *P* <0.001). The difference in hospital LOS between de medium-risk and high-risk group was not significant (*P* = 0.103). The outcome measure “ICU LOS” for each risk group was not significant (*P* = 0.114).

**Figure 4:**
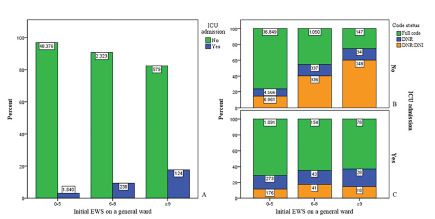
Association between the initial Early Warning Score (EWS) on a general ward and admission to the intensive care unit (ICU). The initial EWS recorded on admission is categorized in low-, medium- and high-risk EWS. A) The percentage and total frequency of ICU admissions categorized into each risk group. Patients admitted to the ICU are depicted in blue, and patients not admitted to the ICU are depicted in green. B) The percentage and frequency of patients not admitted to the ICU categorized into each risk group and subcategorized into code status upon admission to a general ward. C) The percentage and frequency of patients admitted to the ICU categorized into each risk group and subcategorized into code status upon admission to a general ward.

Figure 4A shows the total frequency of ICU admissions for each risk group. Figure 4B/C shows the total frequency of ICU admissions for each risk group, subcategorized into code status. In the high-risk group 579 admissions (83.4%) were not admitted to the ICU (Figure 4B), and 124 admissions (17.6%) were admitted to the ICU (Figure 4C). In the high-risk group 147 patients (25.4%) with a full code were not admitted to the ICU (Figure 4B).

**Figure 5:**
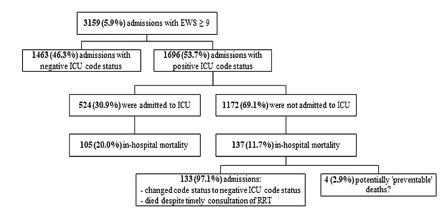
Flowchart of patients with high-risk Early Warning Score (EWS). The “preventable” adverse events group was characterized by patients with a high-risk EWS and a positive ICU code status (to be admitted to the ICU), who were not admitted to the ICU and died during hospital admission.

In 3159 admissions (5.9% of all admissions), a high-risk EWS was recorded at least once during admission. In this high-risk EWS group, 1696 patients (53.7%) were admitted to a general ward with a positive ICU code status (full code or DNR) (Figure 5). In this high-risk group with positive ICU code status, 524 admissions (30.9%) were admitted to the ICU. Of these patients, 105 (20%) died during hospital admission. In the same high-risk group with positive ICU code status, the remaining 1172 patients (69.1%) were not admitted to the ICU. Of these patients, 137 (11.7%) died during hospital admission. Of these 137 patients, 133 patients (97.1%) had their code status changed to a negative ICU code status. The remaining four admissions (2.9%) were patients with at least one high-risk EWS and a positive ICU code status, who were not admitted to the ICU and died during hospital admission.

## DISCUSSION

We found that the initial EWS on a general hospital ward was associated with an increased in-hospital mortality. This result suggests that an elevated initial EWS may help to predict poor outcomes in patients admitted to a general ward. Our study’s major strength is its large study population comprehending 53,180 adult patients admitted to a general hospital ward. Our results correspond with previous studies.^8^,^16^,^17^ Lee et al demonstrated that the National Early Warning Score (NEWS) effectively predicts in-hospital mortality in patients admitted to a general ward. They reported that 18.6% patients with a medium-risk NEWS and 32.6% patients with a high-risk NEWS died during hospital admission.^8^ This result was in agreement with our findings of 10.5% and 25.3%, respectively.

In contrast to our study, Spagnolli et al solely included patients admitted to the emergency department. They reported an incidence of 15.6% medium-risk (NEWS 5–6) and 17.5% high-risk (NEWS ≥ 7) patients compared to our 4.8% and 1.3%, respectively. Despite their higher incidence of medium- and high-risk categories, the in-hospital mortality was 8.2% for medium-risk and 19.2% for high-risk groups compared to our 10.5% and 25.3%, respectively.^17^ This difference may be due to using different EWS systems, different thresholds for risk categories, and a non-similar study population. Comparing the results of studies investigating EWS is difficult because the methodological quality of available studies is diverse.^2^,^9^

The results of studies that have included solely patients admitted to a general ward seem to be more in line with our study.^18^ Van Galen et al considered a Modified Early Warning Score (MEWS) of more than three as a critical score,^19^ which is comparable to our medium-risk EWS. They reported that 7.0% of patients with a critical score and 1.3% of patients with a low-risk MEWS were admitted to the ICU.^1.9^ Their results are in line with our 9.3% and 3.1% ICU admission rates, respectively.

As an elevated initial EWS can help to predict in-hospital mortality, unnecessary deaths could be prevented.^20^,^21^ In our study, these potentially preventable deaths (n = 4, 2.9% of patients with EWS ≥ 9, and <0.01% of the total study population) were identified as patients with at least one high-risk EWS and a positive ICU code status (to be admitted to the ICU), who were not admitted to the ICU and died during hospital admission (Figure 5). Remarkably, other factors were involved in the decision-making process to not admit the patient (with a positive ICU code status and a high-risk EWS) to the ICU. In this group not admitted to the ICU, in-hospital mortality rates were lower than in the group that was admitted to the ICU. This difference in mortality rates could suggest that some patients with high-risk EWS were not admitted to the ICU as they seemed to respond to treatment, although they had a single, high EWS before the intervention. This hypothesis needs to be addressed in further analysis. Although our study showed that EWS could help predict poor outcomes, any EWS should always be interpreted with caution and never can replace clinical judgment.^22^

## LIMITATIONS

A limitation of our study design was its retrospective, single-center nature, which may have allowed bias by indication and residual confounding. Furthermore, MEWS documentation tends to be more complete in patients with a total MEWS of three or more (corresponding with our medium-risk EWS).^23^ By excluding admissions without at least one recorded EWS or with three or more missing variables (in total 29.3% of all admissions), we potentially introduced selection bias. The variables that were missing most frequently in our database were level of consciousness, systolic blood pressure, and use of supplemental oxygen. It could well be that nurses did not appreciate the level of consciousness or the use of supplemental oxygen, because the patient was alert and responsive and did not require supplemental oxygen. In that case, these variables would not have contributed to their total EWS.

## CONCLUSION

Our findings suggest that an initial high-risk Early Warning Score in patients admitted to a general hospital ward is associated with an increased risk of in-hospital mortality, ICU admission, and prolonged hospital length of stay. Therefore, an initial high-risk EWS should raise immediate awareness of the medical and nursing staff. Moreover, close monitoring and precise documentation of the EWS in the electronic health record may facilitate predicting poor outcomes in patients and help to identify patients for whom timely and adequate management may improve outcomes.

## Figures and Tables

**Table 1:** Baseline characteristics of the study population.

	Total	EWS Risk Categories a			P-value b
			
		Low	Medium	High	
EWS		0–5	6–8	≥ 9	
Total admissions, N (%)	53,180 (100)	49,916 (93.9)	2561 (4.8)	703 (1.3)	
Individual patients, N (%)	33,628 (63.2)	32,448 (96.5)	939 (2.8)	241 (0.7)	
Total recorded EWS, N (%)	457,184 (100)	415,489 (90.9)	31,678 (6.9)	10,017 (2.2)	
Females, N (%)	28,233 (53.1)	26,550 (53.2)	1358 (53.0)	325 (46.2)	0.001
Age (year), median [min–max]	68 [18–105]	68 [18–103]	74 [18–105]	76 [18–98]	<0.001
Admission type, N (%)					<0.001
Medical	33,837 (63.6)	30,906 (61.9)	2270 (88.6)	661 (94.0)	
Surgical	19,343 (36.4)	19,010 (38.1)	291 (11.4)	42 (6.0)	
Code status, N (%)					
Full code	39,369 (74.0)	37,940 (76.0)	1204 (47.0)	225 (32.0)	
DNR	5331 (10.0)	4839 (9.7)	380 (14.8)	112 (15.9)	
DNR/DNI	8480 (15.9)	7137 (14.3)	977 (38.1)	366 (52.1)	
Changed code status	1081 (2.0)	882 (1.8)	153 (6.0)	46 (6.5)	<0.001
RRT consultation, N (%)	1400 (2.6)	999 (2.0)	264 (10.3)	137 (19.5)	<0.001

a Based on the initial EWS on a general hospital ward.

b Calculated by Pearson’s chi square or Fisher’s exact test, and a one-way ANOVA where appropriate.

*N*, number of patients; *EWS*, Early Warning Score; *min*, minimum; *max*, maximum; *DNR/DNI*, Do Not Resuscitate/Do Not Intubate; *RRT*, rapid response team.

**Table 2:** Outcomes for the Early Warning Score (EWS) risk categories based on the initial EWS.

	Total	EWS Risk Categories a			P-value b
			
		Low	Medium	High	
EWS		0–5	6–8	≥ 9	
Primary outcome					
In-hospital mortality, N (%)	1205 (2.3)	758 (1.5)	269 (10.5)	178 (25.3)	<0.001
Discharged alive, N (%)	51,975 (97.7)	49,158 (98.5)	2292 (89.5)	525 (74.7)	
Secondary outcomes					
ICU admission, N (%)	1930 (3.6)	1930 (3.6)	1568 (3.1)	238 (9.3)	<0.001
≥1 ICU re-admission, N (%)	76 (0.1)	76 (0.1)	60 (0.1)	10 (0.4)	<0.001
ICU LOS (days), median [Q1–Q3]	2.6 [1.1–5.7]	2.6 [1.1–5.7]	2.5 [1.0–5.4]	2.9 [1.1–7.2]	0.114
Hospital LOS (days), median [Q1–Q3]	4.0 [3.0–7.0]	4.0 [3.0–7.0]	4.0 [3.0–7.0]	7.0 [5.0–11.0]	<0.001

a Based on the initial EWS on a general hospital ward.

b Calculated by Pearson’s chi square or Fisher’s exact test and a one-way ANOVA where appropriate.

*N*, number of patients; *EWS*, Early Warning Score; *ICU*, intensive care unit; *LOS*, length of stay; *Q1–Q3*, first and third quartile.
